# A Design of Experiments Approach for Enhancing Room Temperature Stability of a Lyophilised and Paper-Based Bacterial Cell-Free System

**DOI:** 10.3390/bioengineering12030223

**Published:** 2025-02-22

**Authors:** Tejasvi Shivakumar, Joshua Clark, Alice Goode, Valentine E. Anyanwu, Philip M. Williams

**Affiliations:** Molecular Therapeutics and Formulation, School of Pharmacy, University of Nottingham, Nottingham NG7 2RD, UKpaxaw5@exmail.nottingham.ac.uk (A.G.);

**Keywords:** cell-free protein synthesis, stability studies, design of experiments (DoE)

## Abstract

Centralised cell-based biomanufacturing severely limits applicability in low-resource and extreme environments, where a largely untreated human population is present. Cell-free protein synthesis (CFPS) can surpass many of these limitations, due to its flexibility and low maintenance. After initial optimisation for high-level expression, we conceptualised CFPS platforms composed of lyophilised pellets and cellulose stacks for ease of storage and distribution. The latter platform consisted of lyophilised components on cellulose discs, which were layered and rehydrated to kickstart protein synthesis. Such paper-encompassed reactions were capable of robust expression, where the system can be modulated by simply changing the DNA layer. Using an initial screening design followed by a minimalistic design of experiments approach, we were able to improve the shelf life of lyophilised CFPS at room temperature from <1 week to 100% preservation at month 1. We anticipate that our strategy will enable quicker and more efficient stability optimisation for sustainable applications in all environments.

## 1. Introduction

Cell-free protein synthesis (CFPS) systems offer a versatile, rapid and efficient approach to protein synthesis and have demonstrated production of proteins with high molecular weight: 120 kDa in *E. coli*, 116 kDa in insects and 160 kDa–260 kDa in HeLa and rabbit reticulocyte [1,2,3]. With the maturation of this technology over the past decade, focus has now shifted to instant production of proteins at any desired time and location for utility in fields such as pharmaceuticals (biotherapeutics), diagnostics and biomanufacturing (small molecules and platform compounds). These on-demand systems are required to be compact, easily transportable and stable for suitability in resource-limited settings and/or extreme environments [4,5,6,7]. These requirements are fulfilled, in part, by lyophilised CFPS systems, including ‘just-add-water’ approaches, which offer enhanced stability compared to liquid CFPS [8,9]. Lyophilised CFPS has been shown to retain 79% ± 16% activity at 4 °C and 35.3% ± 7.3% activity at 23 °C for two weeks, surpassing the 56.7% ± 8.9% at 4 °C and no activity at 23 °C reported for liquid extracts [10]. Meanwhile, Pardee et al. showed that freeze-dried cell-free reactions derived from NEB PURExpress™ were stable for up to one year at room temperature, which they exploited for in vitro diagnostics applications [11]. The fact that the reconstituted CFPS system is more stable than cell-derived CFPS systems suggests that the complex mixture of proteins in the latter may accelerate degradation.

Sugars and other molecules are often incorporated as excipients for biotherapeutic formulation and stabilisation, aiding preservation of native protein structure during lyophilisation and at elevated storage temperatures [12]. Sugars exert their mechanism of action by forming a protective shell through water replacement and strengthening intramolecular hydrogen bonds, thereby protecting proteins from denaturation and aggregation [13]. This influence makes sugars a valuable additive in CFPS reactions for stabilisation of proteins under stressful conditions, such as during freeze-thaw, lyophilisation and heat shock. Highly reported candidates for CFPS stabilisation include lactose, trehalose and sucrose and molecular crowding agents such as trimethylglycine, PEG and dextran (e.g., β-cyclodextrin) [10,14,15,16]. Stabilising agents with different mechanisms of action (e.g., sugars and crowding agents) may be supplemented together for a beneficial combinatorial effect. Previously discussed lyophilised CFPS stored at 23 °C for two weeks displayed full preservation through the combined effect of three supplements (PEG, trehalose and trimethylglycine), an improvement from 35.3% ± 7.3% activity with no supplements [10].

In this study, we evaluated the potential of cell-free systems for on-site applications compared to traditional expression methods and explored their feasibility for user-friendly deployment in resource-limited settings. We first described an optimised in-house bacterial cell-free system with capabilities that matched commercial systems such as PURExpress^®^ through overexpression of soluble T7 RNA polymerase. We next experimented with lyophilised pellets and cellulose stacks and evaluated their impacts on CFPS yield and stability. As our results indicated a loss of stability over time, we turned our attention to screening various supplements that have had known positive effects in the past. Previously unknown effects of the supplements on CFPS kinetics were discovered, and further insights into short-term stability were obtained. Taking inspiration from previous simplistic design of experiments (DoE) type approaches for other applications, we opted for a similar approach to identify combinations of high-performing supplements [17]. One such DoE combination from our minimalistic design led to 100% preservation of CFPS stability at room temperature for one month. Based on the work carried out here compared to elsewhere, we found that optimisation of supplements must be carried out for each unique CFPS system due to variability in stability between research groups. Our short-term screening and design of experiments approach describes a minimalistic and cost-effective method for improvement in CFPS stability without time- and resource-intensive studies, which can be easily transferable to other CFPS systems. These outcomes highlight the practical relevance of the system, offering a viable alternative to traditional protein expression methods, making it suitable for point-of-care diagnostics, rapid protein prototyping outside of laboratory settings and synthetic biology solutions. By incorporating lyophilisation, the stability and portability of CFPS reagents are significantly improved, enabling easier storage and transport for field deployment. Moreover, the system provides an affordable and sustainable design for researchers and industries, helping to reduce costs and the carbon footprint associated with cold-chain storage and transport. These attributes bring on-demand biomanufacturing and biosensing one step closer to practical utilisation in diverse settings, including resource-limited regions.

## 2. Materials and Methods

### 2.1. Strains and Growth Condition

*Escherichia coli* BL21 Star (DE3) [*E. coli* BL21 Star (DE3)] was transformed with plasmid pAR1219, purchased from Sigma Aldrich (TargeTron™; Darmstadt, Germany), to obtain recombinant strain *E. coli* BL21 Star (DE3)-pAR1219, which was used for all the extract preparation except where otherwise mentioned. The superfolder green fluorescent protein (sfGFP) gene fragment sf*gfp* was obtained from pBAD24-sf*gfp*x1 (Addgene plasmid #51558; Watertown, MA, USA), amplified by PCR and assembled using NEB HiFi DNA into a pET20b vector with a 6xHistidine tag to obtain pET20b-sf*gfp*-6xHis (the template DNA for CFPS, except otherwise mentioned) and cloned into *E. coli* NEB5α. In addition, empty pET20b (the control DNA for CFPS, except where otherwise mentioned) was cloned into *E. coli* NEB5α. Plasmid DNA (pET20b-sf*gfp*-6xHis and pET20b) was extracted from *E. coli* NEB5α—pET20b-sf*gfp*-6xHis and *E. coli* NEB5α—pET20b, grown overnight in Luria–Bertani (LB) broth, supplemented with 100 µg/mL ampicillin at 37 °C and 250 revolution per minute (RPM). A Qiagen maxiprep kit was used to perform plasmid extraction following the manufacturer’s protocol, and DNA was eluted in nuclease-free water. Final DNA concentration was measured using a Nanodrop™ 2000 spectrophotometer (Thermo Fisher Scientific, Wilmington, DE, USA).

### 2.2. Cell-Free Extract Preparation

The protocol for cell-free extract preparation was adapted from a previously established and optimised workflow for *E. coli* BL21 Star (DE3) [18]. Our transformed *E. coli* BL21 Star (DE3)-pAR1219 strain was first cultured overnight in LB broth containing 100 µg/mL ampicillin. This overnight culture was then used to inoculate 750 mL of 1× YTPG media (10 g/L yeast extract, 16 g/L tryptone, 7 g/L KH_2_PO_4_, 3 g/L K_2_HPO_4_, 0.4 M glucose and 5 g/L NaCl) supplemented with 100 µg/mL ampicillin in 2 L baffled shake flasks, starting at an OD_600_ of 0.1. The culture was incubated at 37 °C and 250 RPM until an OD_600_ of 0.8 was reached, at which point T7 polymerase expression was induced with 1 mM IPTG (isopropyl β-D-1-thiogalactopyranoside). Following induction, the cells were allowed to grow until reaching an OD_600_ of 4.0 and were then harvested by centrifugation at 7000 RPM and 4 °C for 5 min. The cell pellet was washed three times and finally resuspended in S30A buffer (50 mM Tris, 14 mM magnesium glutamate, 60 mM potassium glutamate, pH 7.8). The suspension was supplemented with a protease inhibitor (Roche; Burgess Hill, UK) and 2 mM dithiothreitol. Cell-free extract was prepared by sonicating the cell suspension on ice using a Bandelin Sonopuls HD 2070 ultraprobe Digital Sonicator (Bandelin Electronics, Berlin, Germany) at 70% amplitude, applying four cycles of 45 s each with one-minute rest intervals (totalling 3 min). After sonication, the lysate was supplemented with an additional 2 mM dithiothreitol and clarified by centrifugation at 18,000× *g* for 10 min at 4 °C. A run-off reaction was then performed at 37 °C and 250 RPM for one hour. Finally, the extract was obtained by centrifuging the run-off reaction at 12,000× *g* for 10 min at 4 °C, flash-freezing the supernatant in liquid nitrogen and storing it at −80 °C until use. Protein concentration was determined using a Micro BCA™ Protein Assay Kit (Thermo Fisher Scientific, Wilmington, DE, USA).

### 2.3. Cell-Free Protein Synthesis

In-house CFPS reactions were set up on ice as previously described [18]. Briefly, reactions were incubated at 37 °C and 180 RPM with the composition outlined in Table 1, whereas kinetic sfGFP fluorescence assays were set up in black, flat-bottom 96-well assay plates (Thermo Fisher Scientific). The reaction mix varied according to the experimental design, where the volume of water or stock concentration was adjusted where necessary and master mixes were prepared for DoE experiments to reduce variation in the incubation times for each condition. Supplements described in the stability studies were prepared as 1 M stocks in nuclease-free water and stored at 4 °C for up to a month. Polyethylene glycol (PEG; molecular weight 600 g/mol, 6000 g/mol and 8000 g/mol) was prepared as a 50% *w*/*v* stock, diluted in nuclease-free water.

Lyophilised cell-free reactions were prepared in 1.5 mL Eppendorf tubes and flash-frozen in liquid nitrogen immediately after setup [14]. The reactions were placed in a freeze-dryer prechilled to −60 °C and were lyophilised overnight under vacuum (<120 mTorr) using a VirTis Benchtop Lyophiliser Sentry 2.0 (SP Industries, Stone Ridge, NY, USA).

Analytic techniques for determining cell-free sfGFP synthesis included time course and endpoint analyses. For kinetic time course analysis, fluorescence was measured continuously every 10 min over a period of 12 to 24 h using a TECAN Spark^®^ Multimode Microplate reader (Männedorf, Switzerland). For timepoint analysis, after incubating cell-free reactions for four hours in a shaker incubator at 180 RPM, both fluorescence readings and protein samples were collected using a NanoDrop™ 3300 Fluorospectrometer (Thermo Fisher Scientific, Wilmington, DE, USA). In both cases, sfGFP was detected with an excitation wavelength of 485 nm and an emission wavelength of 510 nm.

### 2.4. CFPS on Cellulose Stacks

Lyophilisation of aqueous components onto paper discs serves as a versatile scaffold for the assembly of CFPS reactions in a modular fashion. Preliminary experiments employed toilet paper; however, due to the absence of controlled and verified properties, an exploration for an alternative substrate ensued. Seven paper types with validated laboratory applications were evaluated: toilet paper (2-ply Versatwin toilet roll, 125 m length, 90 mm width); Whatman number 1 filter paper; Whatman number 5 filter paper; cellulose triacetate (Selectophore^®^, Gillingham, UK); Hydrosart membrane filter (Sartorius, Göttingen, Germany); polyethersulfone membrane (Sartorius, Göttingen, Germany); nitrocellulose Membrane, 0.45 µm (BioRad, Watford, UK).

Autoclaved and hole-punched paper discs of 5 mm diameter were added to either extract supplemented with RNAse inhibitor, DNA template or energy and buffers, with final concentrations outlined in Table 1. Freeze-drying was carried out by flash-freezing paper containing tubes in liquid nitrogen and lyophilised overnight. Rehydration was carried out by layering each of the three papers (one each with extract, DNA and buffers) in one tube with the addition of 40 µL S30A buffer. Reactions were incubated at 37 °C and 180 RPM overnight, unless otherwise specified. sfGFP expression was determined as described previously.

### 2.5. Design of Experiments (DoE)

A two-factor factorial design was employed to investigate the relationship between sfGFP fluorescence and four independent variables (supplement final concentration in cell-free reaction): maltodextrin (mM), β-lactose (mM), PEG (MW), and PEG (%). The experimental design was generated using the MiniTab software (Version 21.3) ‘general full factorial design’ tool. The study consists of 8 experimental runs including 3 repeats for each additive combination and a control run with the absence of the 4 test factors. Randomisation of the run order was performed using a seed generated by the software. Samples were prepared, lyophilised and rehydrated as described previously with the addition of the run-specific factors. The fluorescence of the samples was used as the response variable, as the standard proxy for sfGFP yield. MiniTab was further used for factorial design analysis in a stepwise selection method to automatically select main effects and/or two-factor interactions using a factorial regression model and factorial ANOVA. The response variable (fluorescence) was regressed against the main effects and two-way interactions of the factors. Coded coefficients were derived to facilitate comparisons between factors.

### 2.6. Stability Studies

Accelerated stability studies of lyophilised CFPS were designed in accordance with ICH guidelines, taking into account the required temperature, humidity and time conditions. A sufficient number of 50 µL stability samples were prepared and lyophilised in 1.5 mL Eppendorf tubes. Each sample was labeled with its batch number, storage temperature, preparation date and sampling time point before being placed either in a stability oven (40 °C ± 2 °C/75% RH ± 5% RH) or in a cold room (4 °C). For experiments at room temperature (approximately 19 °C), samples were kept on the lab bench alongside a temperature monitor (Thermopro TP49; Duluth, GA, USA). To assess stability at predetermined time points, each sample was first rehydrated with S30A buffer to achieve 80% of the total reaction volume, then incubated at 37 °C for 16 h at 180 RPM, followed by endpoint fluorescence analysis.

### 2.7. SDS-PAGE and Western Blotting

For analysis of protein expression, SDS-PAGE (sodium dodecyl sulphate polyacrylamide gel electrophoresis) and western blotting techniques were employed. Samples were denatured with SDS containing sample buffer and separated on a 12% TGX (Tris/Glycine/SDS) gel. Following electrophoresis, gel was stained using InstantBlue™ Coomassie stain (Abcam; Cambridge, UK). For western blot analysis, the gel was instead transferred onto a nitrocellulose membrane (BioRad) using a BioRad Trans-Blot Turbo Transfer system, and the SuperSignal™ West HisProbe kit (Thermo Scientific™; Wilmington, DE, USA) was used to obtain an image of the blot (BioRad Gel Doc; Watford, UK) following chemiluminescent detection.

### 2.8. Statistical Analysis

All graphs and statistical analyses were created and conducted in GraphPad Prism version 10.0.0 for Windows 10, GraphPad Software, Boston, MA, USA. Unless otherwise specified, significance was determined between conditions using unpaired, two-tailed *t*-tests. For stability studies, one-way ANOVA followed by Tukey’s multiple comparisons test was performed for comparing supplemented CFPS stored for various amounts of time against 0-week and unsupplemented/fresh CFPS. We tested the assumption of homogeneity of variance using the Brown–Forsythe test, and normality of residuals was confirmed using the Shapiro–Wilk test. We also report 95% confidence intervals for group means and differences to provide a clearer picture of the variability and reliability of the estimates. The adjusted *p*-value denotes the significance of preservation, where *p* < 0.0001 is marked ****, 0.0001 to 0.001 by ***, 0.001 to 0.01 by **, 0.01 to 0.05 by * and ≥0.05 by *ns* (not significant).

## 3. Results

### 3.1. Development and Optimisation of Bacterial Cell-Free System

Wild-type (WT) *E. coli* BL21 Star (DE3) was selected as the primary candidate for cell-free extract preparation due to its robust protein expression capabilities driven by T7 polymerase, rapid growth and reaction times and established history with CFPS [18]. Effective CFPS requires high levels of soluble, active T7 polymerase, which is often supplemented externally—an approach that increases both cost and processing time. To overcome this limitation and enhance CFPS performance, we transformed WT *E. coli* with pAR1219 to boost its endogenous T7 RNA polymerase production. Our observations indicated that the transformed strain’s growth rate was comparable to that of the WT (Supplementary Figures S1–S5). Additionally, SDS-PAGE analysis detected T7 polymerase expression in both *E. coli* BL21 Star (DE3)-pAR1219 and the WT within two hours after induction, with notably stronger expression in the IPTG-induced *E. coli* BL21 Star (DE3)-pAR1219. Although a similar expression pattern was observed five hours post-induction, T7 polymerase was predominantly found in the insoluble fraction at that time (Supplementary Figure S5). These results suggest that cells used for cell-free extract preparation must be harvested by 2 h post-induction at 37 °C. This correlates well with the mid-exponential phase of growth and timings of harvest reported in other methods [19,20,21,22].

Successful CFPS relies on maintaining optimal concentrations of various components in the reaction mixture. Magnesium ions (Mg^2+^) act as essential cofactors for many translation enzymes, contribute to ribosome assembly and seem to offer a protective effect during protein lyophilization [23,24,25]. One of the key objectives of this study was to identify an optimal magnesium glutamate (Mg-Glu) concentration, as previous studies have particularly emphasised the importance of its optimisation for each new cell-free extract preparation [23,26,27].

**Figure 1:**
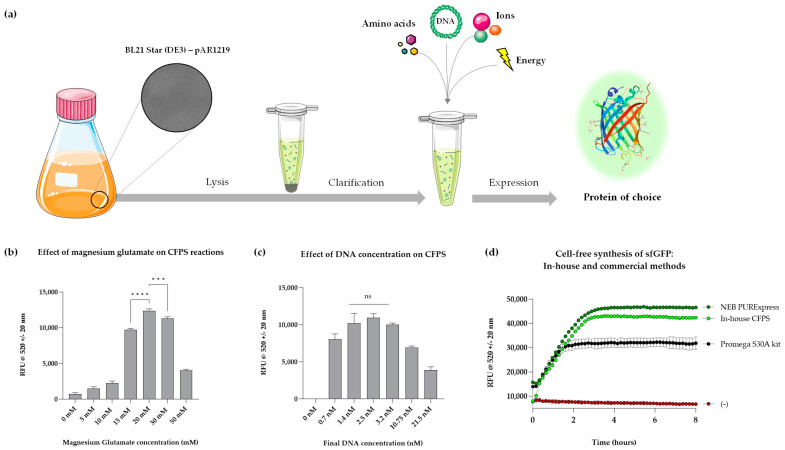
(**a**) Cell-free workflow demonstrating the use of E. coli BL21 Star (DE3)—pAR1219 cells to create cell-free extract, which can then be supplemented with amino acids, DNA, ions and energy to synthesise a protein of choice. (**b**) Graph of sfGFP fluorescence from cell-free reactions at varied concentrations of Mg-Glu after 4 h of incubation. (**c**) Graph of sfGFP fluorescence from cell-free reactions at varied concentrations of DNA after 4 h of incubation. Fluorescence was normalised with negative control reactions (pET20b). (**d**) A comparison of time course analysis of sfGFP production (mg/mL) in in-house and commercial CFPS systems (PURExpress™; Frankfurt, Germany and Promega S30A kit; Southampton, UK for circular DNA). *p* value > 0.05 = ns, *p* < 0.001 = *** and *p* < 0.0001 = ****; experiments were performed in triplicates (n = 3); data are shown as means ± SD.

A series of Mg-Glu concentrations, ranging from 0 mM to 50 mM in 5 mM increments, was evaluated through time course analyses. It was evident that the absence of Mg-Glu inhibited protein synthesis, while samples containing 20 mM Mg-Glu produced the highest fluorescence signal (Figure 1b). Concentrations above 20 mM appeared to suppress protein synthesis, which is consistent with previous findings [28,29]. Therefore, 20 mM Mg-Glu was chosen for all subsequent experiments. Under these optimised Mg-Glu conditions, a range of DNA concentrations was also tested to determine the level that yielded the highest fluorescence. Endpoint fluorescence increased with rising DNA concentrations up to 2.5 nM, after which the yield declined as DNA levels increased further (2.5 nM–21.5 nM; Figure 1c). Consequently, 2.5 nM DNA and 20 mM Mg-Glu were established as the optimal conditions for further studies.

The performance of this optimised reaction mixture was compared with commercial CFPS systems, including PURExpress^®^ (manufactured by NEB, Frankfurt, Germany) and the *E. coli* S30A Extract System for Circular DNA (manufactured by Promega, Southampton, UK). PURExpress^®^ is a well-established, highly efficient reconstituted system composed of purified enzymes from *E. coli* BL21 cells, whereas the Promega system utilises crude bacterial cell extract from a strain lacking OmpT endoproteinase and ion protease. Both systems are among the most popular CFPS kits available [30,31,32]. Benchmarking was carried out by monitoring sfGFP synthesis over time under identical conditions (50 µL reactions incubated at 37 °C, as recommended by both manufacturers). Time course analyses revealed that the sfGFP synthesis trend of our in-house CFPS system closely paralleled that of PURExpress^®^ and produced a higher yield than the Promega system (Figure 1d). Although PURExpress^®^ yielded the highest overall sfGFP levels, our in-house system’s performance was significantly better than Promega’s and reached approximately 90% of the yield achieved by PURExpress^®^. Since both our in-house and the Promega systems are based on similar strains, the increased CFPS yield can be attributed to enhanced T7 RNA polymerase levels and the optimised concentrations of Mg-Glu and DNA. Further optimisation of other reaction components, such as the energy mix, may lead to even greater outputs.

### 3.2. On-Demand Synthesis Enabled by Lyophilised and Paper-Based Formats

Lyophilisation, or freeze-drying, is a widely used technique for creating solid protein formulations to enhance stability. While it offers numerous benefits, it also presents several challenges that have been extensively reviewed over the past few decades [33,34,35]. Unsurprisingly, lyophilisation has also been applied to cell-free systems, preserving both extracts and complete cell-free reactions. This not only improves stability compared to standard liquid formats but also facilitates easy transportation and rehydration, features that are highly desirable for on-site and on-demand applications [9,14,36,37,38].

**Figure 2:**
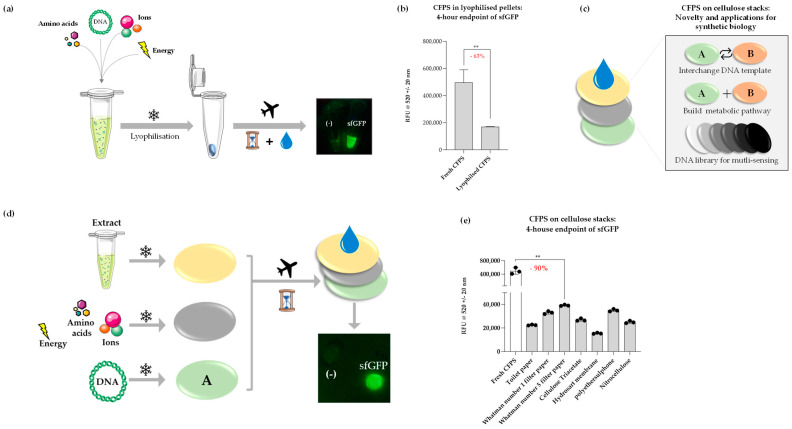
(**a**) Schematic of CFPS in lyophilised format; fluorescence was detected following rehydration of lyophilised pellets containing sfGFP DNA (‘sfGFP’) and no template (‘-’) imaged under a blue-light transilluminator. (**b**) Comparative analysis of endpoint sfGFP signal between freshly prepared and lyophilised CFPS samples normalised to negative control samples. (**c**) Illustration of the novelty of cellulose stack format. The DNA layer can be easily interchanged for biomanufacturing or biosensing applications and is particularly useful for multiple sensing (e.g., heavy metal contamination) applications using the same sample in the field. (**d**) Schematic of CFPS on cellulose stacks separated onto three cellulose discs, corresponding to DNA, cell-free extract and energy/buffer constituents, respectively. Fluorescence was detected following rehydration of cellulose stacks containing sfGFP DNA (‘sfGFP’) and no template (‘-’) imaged under a blue-light transilluminator. (**e**) Comparative analysis of endpoint sfGFP signal following rehydration of lyophilised cellulose stacks on various paper substrates normalised to negative control samples, shown alongside a freshly prepared reaction. *p* < 0.01 = **; experiments were performed in triplicates (n = 3); data are shown as means ± SD.

We first evaluated the ability of CFPS to be preserved in this manner by lyophilising cell-free reactions overnight (Figure 2a). The impact of this procedure was studied by measuring cell-free sfGFP synthesis immediately following rehydration of the lyophilised pellets. sfGFP fluorescence from samples that underwent lyophilisation was reduced by ~63% when compared to freshly prepared CFPS reactions (Figure 2b). Despite this, a strong fluorescence signal was observed when samples were placed under a blue-light transilluminator. We applied this lyophilisation method to develop a tunable paper-based CFPS platform, primarily for biosensing applications similar to others described for educational kits and portable applications [11,39,40]. The intention behind the paper-based platform is to create a flexible and interchangeable approach, where the DNA component may be easily changed to produce any therapeutics at the point of care, that is also easy to adapt for high-throughput sensing applications (Figure 2c). The key components of this platform were three cellulose discs individually lyophilised with the following CFPS components: cell extract, energy and DNA (Figure 2d). The system was designed such that each lyophilised disc could be layered and rehydrated in a modular fashion to kickstart protein synthesis. Seven types of paper were tested for their ability to support lyophilised CFPS components: toilet paper; Whatman number 1 filter paper, 11 µm; Whatman number 5 filter paper, 2.5 µm; cellulose triacetate (Selectophore^®^); Type 144 Hydrosart Ultra Filtration Membrane Discs, 10 kDa MWCO (Sartorius™); polyethersulfone membrane, 0.1 µm, 10 kDa MWCO (Sartorius™) and nitrocellulose membrane, 0.45 µm (BioRad).

sfGFP signal from rehydrated cellulose stacks ranged widely depending on the type of paper used (Figure 2d). However, a significant reduction (~90%) was noted when compared to freshly prepared CFPS reactions, which may perhaps be attributed to the variation in component concentration retained on each layer. Whatman number 5 filter paper reported the highest signal of all materials tested, whereas Hydrosart membrane reported the lowest. Polyethersulphone was initially chosen due to its existing applications in hollow fiber bioreactors and its ability to adsorb proteins efficiently [41]; CFPS reactions supported on this polymer produced the second-highest signal. Evidently, the material properties affect the stability and efficiency of CFPS components immobilised on a stack support. Although it is difficult to draw specific conclusions aboutthe effect of each material due to non-disclosure of all materials by the commercial suppliers, we anticipate the highly cellulosic materials supported the reactions well and envisage further investigation by scanning electron microscopy, which could shed light on the influence of parameters such as pore size and molecular interaction on CFPS supported on cellulose stacks. Nevertheless, Whatman number 5 filter paper was identified to be an efficient and cost-effective support for lyophilised CFPS, which contains potential for further improvement in yield with the addition of lyoprotectants. Yet, the signal, as it is, from this cellulose type is adequate for many applications involving on-site biosensing, for example, with easy visualisation of the signal under a blue-light transilluminator, as pictured (Figure 2d).

### 3.3. Lyophilised Cell-Free Systems Perform Poorly in Pharma-Grade Stability Tests

**Figure 3:**
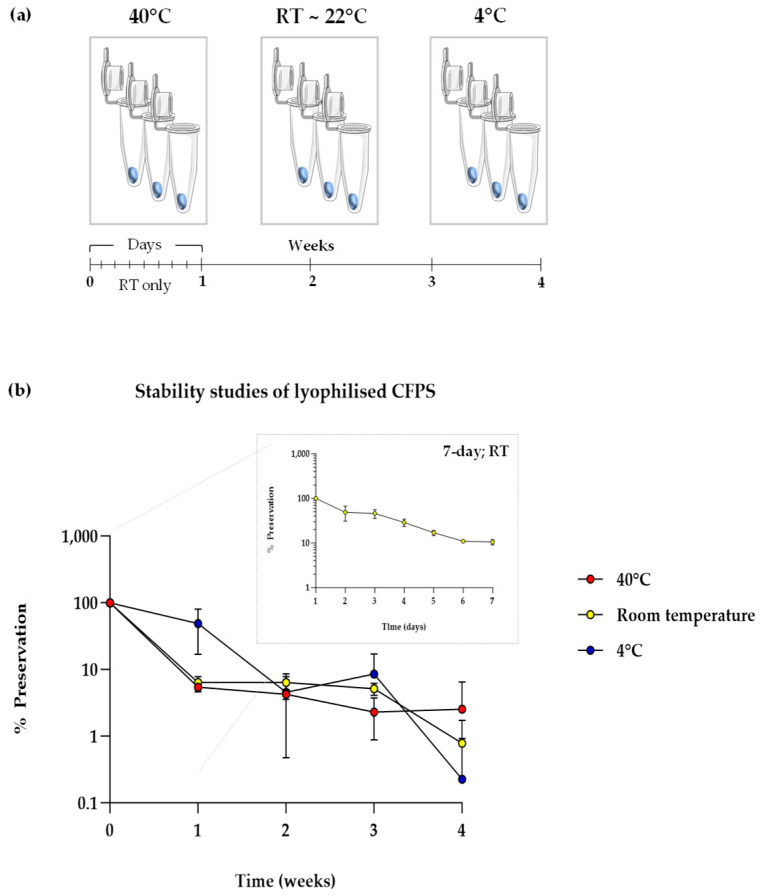
Stability studies of lyophilised CFPS. (**a**) Schematic illustrating the experimental plan for a 4-week stability study at a range of temperatures, with readings taken every day per week for samples stored at room temperature (RT). (**b**) CFPS in lyophilised pellets format after storage for 1 month at 40 °C, RT (~22 °C) and 4 °C with data points shown for the first seven days for samples RT (box). sfGFP fluorescence was normalised to the negative control (pET20b reactions), and % preservation was calculated by normalising obtained fluorescence values to day 0/week 0 fluorescence values, i.e., samples with no storage; experiments were performed in triplicates (n = 3); data are shown as means ± SD.

A well-known bottleneck in cell-free synthetic biology is the need to store cell-free components from −20 °C to −80 °C, owing to their degradation at elevated temperatures. A similar trend for lyophilised cell-free systems has been reported previously; however, the rate of degradation is slower than in aqueous systems, which is attributed to the much-reduced molecular motions in solid state [14,42]. To test if this applied to our lyophilised CFPS system, lyophilised pellets containing sfGFP DNA template were subject to stability studies at three temperatures, namely, 40 °C, room temperature (RT; average 19 °C) and 4 °C (Figure 3a). Samples stored at a set temperature were rehydrated and surveyed for cell-free sfGFP synthesis at five time points from day 0 (immediately after lyophilisation) to weeks 1, 2, 3 and 4. Lyophilised pellets stored at RT were further sampled from days 1–7.

In all temperatures tested, stability dropped significantly over four weeks (Figure 3b). All samples stored at 40 °C and RT lost activity just one week after storage. Activity of samples at 4 °C was significantly higher at one week compared to the former; however, a high variation was observed. This suggests that lowering the temperature may enhance stability in the short-term, owing to higher survival rates of proteins and energy components in lyophilised CFPS. Furthermore, a 50% drop in stability was observed just after one day of storage at room temperature, which reduced to 75% by days 4 and 5. By day 7 (1 week), all stability was lost, which was the observed result for all succeeding data points and temperatures. In summary, the shelf-life of lyophilised CFPS was found to be <1 week and requires substantial improvement for utility. This is, however, not an uncommon result, as similar diminishing trends in stability have been reported previously, where no stability was observed in uncomplemented CFPS after just one day of storage at 37 °C [43]. On a similar note, it was discussed that the cell-free extract degraded faster than the other buffer components. Therefore, innovative solutions for enhancing protein stability may aid any efforts in improving the long-term stability of lyophilised CFPS systems, which was our next focus.

### 3.4. Effects of Various Lyoprotectants and Stabilisers on CFPS Kinetics and Stability at Room Temperature

Sugars are often incorporated as excipients in biotherapeutic stabilisation for their ability to preserve native protein structures. During lyophilisation, these are preserved in an amorphous state [12]. Indeed, most reported candidates in literature for CFPS shelf-life stabilisation are carbohydrates such as sucrose, trehalose and lactose, but also other molecular crowding agents such as trimethylglycine and dextrins (e.g., β-cyclodextrin) [10,14,15,16]. We initially sought to study the effect of the select protectants sucrose, trehalose, α-lactose, β-lactose, β-cyclodextrin and maltodextrin (MDX) on cell-free reaction kinetics prior to their impact on stability. Reaction kinetics provides insight into the effects of a particular supplement, which in turn is a key parameter that is required to adapt system design according to the intended application. An example would be rapid point-of-care CFPS, whether it is necessary for biomanufacturing or diagnostics, where faster kinetics are desirable. Additionally, no kinetic data are currently available for several of the supplements we investigated.

**Figure 4:**
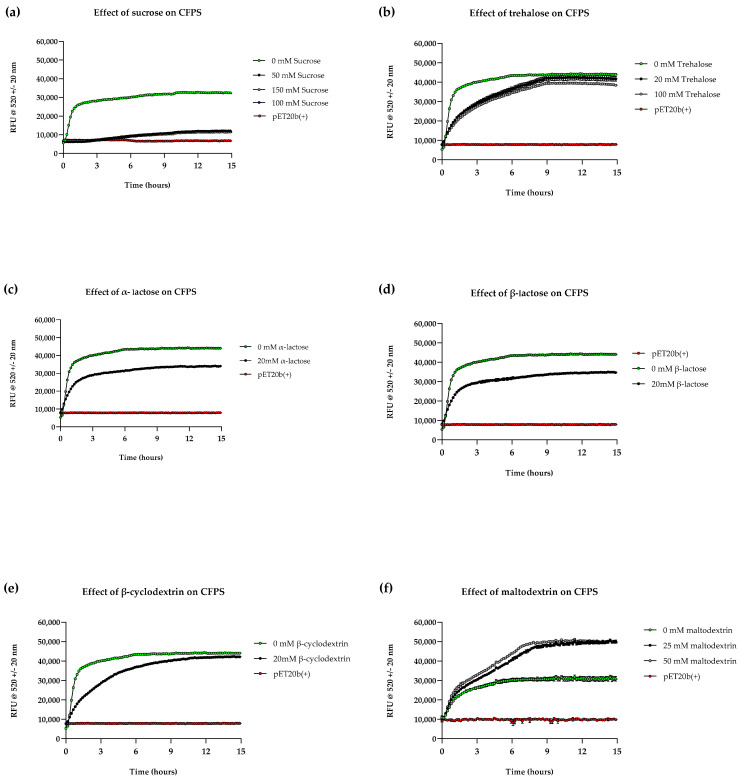
Impact of various supplements on CFPS. Time course analysis showing expression of sfGFP from in-house cell reactions in aqueous format supplemented with (**a**) sucrose, (**b**) trehalose, (**c**) α-lactose, (**d**) β-lactose, (**e**) β-cyclodextrin and (**f**) maltodextrin, compared with unsupplemented reactions (0 mM) and negative control (‘pET20b+’) at 37 °C. Experiments were performed in triplicates (n = 3); data are shown as means ± SD.

Time course analyses were conducted to monitor sfGFP synthesis upon supplementation at various concentrations that were selected based upon previously published work, serving as an initial starting point; ranges of concentrations were tested for some candidates where advised [10,14,15,16]. The first observation was that cell-free kinetics and yield were notably different in all supplemented reactions when compared to unsupplemented reactions (Figure 4). Reactions supplemented with sucrose, trehalose, and β-cyclodextrin displayed significantly lower reaction rates, where synthesis reached saturation at ~8 h, whereas unsupplemented reactions saturated 3–4 h after commencement. Reactions supplemented with α/β-lactose displayed dynamics similar to unsupplemented reactions; however, sfGFP fluorescence was compromised. Most supplemented reactions had significantly lower signals, with sucrose displaying the lowest (30% of the unsupplemented reaction) and trehalose and β-cyclodextrin displaying the highest (90 and 95% fluorescence of unsupplemented reaction, respectively). The low rates of reaction and subsequent yields could be attributed to molar ratios of the supplement and proteins in the cell-free extract, as high affinity of supplements to water reduces molecules available for protein interaction [44]. This may explain why a concentration-dependent decrease in yield was observed in reactions supplemented with trehalose and sucrose (e.g., 100 mM trehalose supplement had reduced fluorescence compared to a 20 mM supplement).

On the other hand, MDX outperformed the unsupplemented reaction, suggesting a yield that is 1.5 times that of the latter (Figure 4f). As for the reaction kinetics, the initial reaction rate matched that of the unsupplemented reaction, but it was followed by another slower exponential phase in both concentrations tested. By 16 h, there was no significant difference between 25 mM and 50 mM MDX-supplemented reactions. The increase in sfGFP synthesis may be explained by the role of MDX as a secondary energy source. The second exponential phase of the reaction can be attributed to the slow metabolism of MDX and its phosphate scavenging properties, as accumulation of inorganic phosphate typically leads to reaction saturation [45,46].

**Figure 5:**
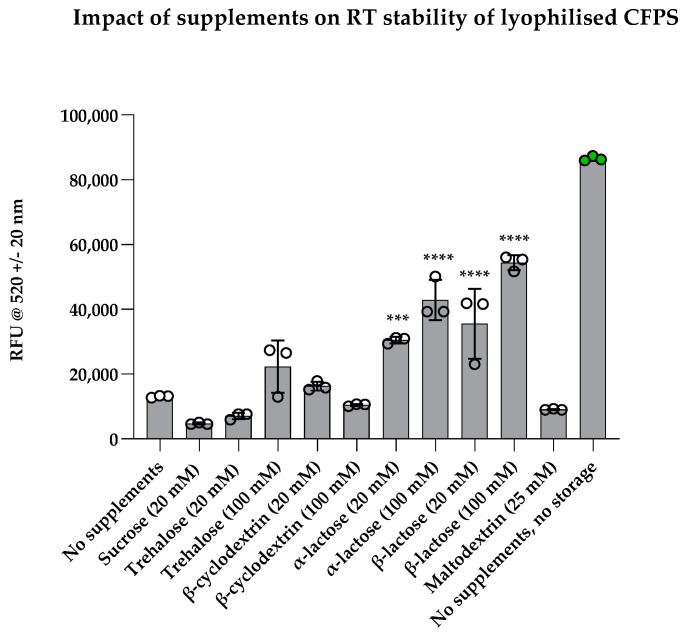
Effect of supplements on short-term stability. Graph shows sfGFP fluorescence following rehydration of lyophilised cell-free pellets with S30A buffer after storage at RT for five days. Experimental samples were normalised to the negative control (pET20b+) and shown alongside the unsupplemented control (‘no supplements’) and a positive control (‘no supplements, no storage’). Reactions with significantly high stability compared to unsupplemented reactions are indicated with significance values above bars; experiments were performed in triplicates (n = 3); data are shown as means ± SD; *p* < 0.001 = *** and *p* < 0.0001 = ****.

We then utilised a short-term stability screening approach, which significantly accelerated the time taken to identify the best CFPS stabilisers without extensive long-term stability studies. Preservation of stability was evaluated by measuring sfGFP fluorescence after rehydrating supplemented reactions following five days of storage at RT, where a fresh CFPS reaction without any supplements or storage served as a positive control. Sucrose was chosen for short-term stability testing despite significant reduction in yield (in Figure 4a), owing to its stability conferring properties previously reported in literature [16]. The addition of sucrose, trehalose, β-cyclodextrin and maltodextrin had no significant difference when compared with unsupplemented reactions (Figure 5). On the other hand, lactose had significantly higher stabilising properties. When preserved with 20 mM α-lactose, activity of CFPS was significantly higher than unsupplemented CFPS (*p* = 0.0005). Higher concentration of α-lactose (100 mM) further increased activity significantly (*p* < 0.0001). Both 20 mM and 100 mM β-lactose significantly preserved CFPS activity (*p* < 0.0001). The mean fluorescence values for reactions preserved with 20 mM α-lactose were lower than that of β-lactose, and similarly, 100 mM α-lactose was lower than that of β-lactose. This difference perhaps arises from higher solubility and higher compaction of β-lactose (due to the presence of more spherical particles and a higher degree of fragmentation) [47]. Alternatively, α-lactose may be spatially repulsing proteins in the cell-free extract due to steric hindrance caused by the secondary hydroxyl group in its chemical structure.

Collectively, these findings led to the identification of β-lactose as an ideal candidate for short-term preservation of lyophilised CFPS. However, the lack of preservation observed in sucrose and maltodextrin-supplemented reactions was not in agreement with previously reported findings [16]. Warfel et al. found that maltodextrin and sucrose enhanced CFPS stability over 4 weeks at 37 °C when supplemented with three concentrations: 30 mg/mL, 60 mg/mL and 100 mg/mL; however, lactose was not tested [16]. These contradictions suggest that optimisation of lyoprotectants and cryoprotectants should perhaps be optimised for each unique CFPS system, akin to the differences in optimal glutamate concentrations reported between groups working on similar CFPS systems.

### 3.5. DoE Strategy Enables the Identification of Combinations of Protectants That Enhance Room-Temperature Stability of Lyophilised Cell-Free System

Sugars clearly play an important role in stabilising cell-free components at room temperature and improving CFPS yields, based on the one-factor-at-a-time screening approach employed so far. However, their combinatorial effects and impact of molecular crowding are still unknown. Based on yield-improving effects of maltodextrin (Figure 4f) and stability-conferring properties of β-lactose (Figure 5), we hypothesised that their combinatorial effect may lead to longer-term CFPS stability. Therefore, we selected four main factors, MDX, β-lactose, PEG % and PEG molecular weight (MW), and utilised a design of experiments (DoE) approach to investigate their effects on the stability and productivity of CFPS. This approach was adopted to systematically investigate the effects of interactions between factors, and a two-level design was initially adopted to minimise sample size while maintaining a sufficient evaluation of the factors in just eight experimental runs, plus a control group with no additives (Table 2).

**Figure 6:**
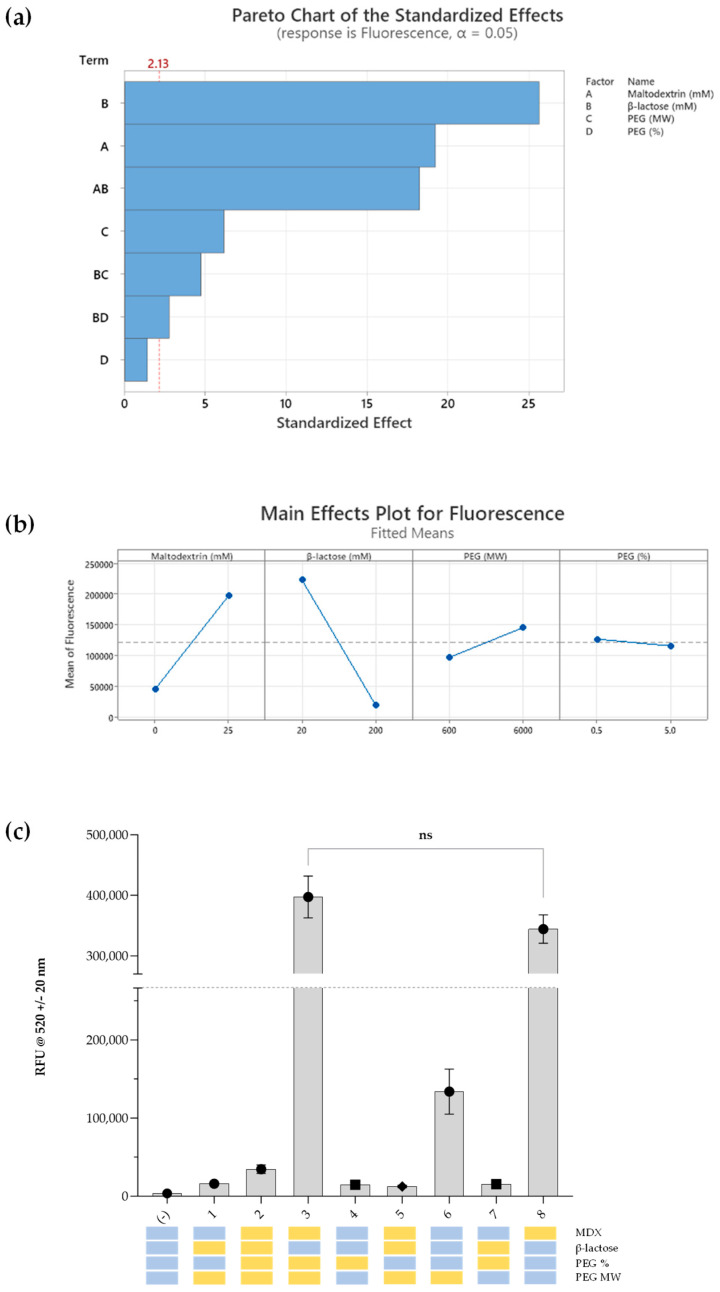
(**a**) Pareto chart of standardized effects for fluorescence response. This chart illustrates the standardized effects of factors and interactions derived from a factorial regression model on fluorescence. Each bar represents the standardized effect of a specific factor or interaction, arranged in descending order. The cut-off line, (---2.13), represents the significance threshold based on α-level = 0.05. Factors and interactions above this line are considered statistically significant (*p* < 0.05). (**b**) Main effects for the fluorescence plot showcase the impact of individual factors on the fitted means of fluorescence derived from the factorial regression model. Vertical bars indicate the magnitude and direction of the effect for each factor, providing a visual representation of their influence on fluorescence. (**c**) Short-term stability studies. Graph showing sfGFP fluorescence following rehydration of lyophilised cell-free pellets with S30A buffer after storage at RT for five days. Experimental samples were supplemented with the variables shown below the data points marked in yellow blocks (high concentrations or presence of MDX) and blue blocks (low concentrations and absence of MDX). ‘pET20b+’ corresponds to the negative control, and the positive control (fresh CFPS in aqueous format without storage) is indicated by the grey dotted line (RFU = 275,000). Experiments were performed in triplicates (n = 3); data are shown as means ± SD; *p*-value > 0.05 = ns.

Eight DoE sample groups were applied using factorial regression modelling to endpoint sfGFP fluorescence after rehydration following RT storage for five days (Figure 6). A substantial positive impact on sfGFP synthesis was evident with the addition of maltodextrin (coefficient: 13125 [*p* < 0.05]; Figure 6a,b). Similarly, lower concentrations of β-lactose exhibited a significant increase in sfGFP fluorescence (coefficient: −220.4 [*p* < 0.05]), and formulations containing high molecular weight PEG demonstrated a smaller yet statistically significant fluorescence increase (coefficient: 17.47 [*p* < 0.05]). The percentage of PEG, however, did not yield a statistically significant impact on fluorescence (Figure 6a). A notable interaction was observed between MDX and low β-lactose concentrations (coefficient: −64.09 [*p* < 0.05]). The goodness of fit for the model was assessed through standard deviation of residuals and R^2^ values. The model demonstrated high explanatory power (R^2^ = 99.00%) with adjusted and predicted R^2^ values of 98.53% and 97.75%, respectively, and the model exhibited overall significance (F value = 212.15, *p* < 0.05).

Our analyses facilitated the discovery of two high-performing formulations, both featuring MDX (25 mM) and low concentrations of β-lactose (20 mM) (Figure 6c). These formulations not only exhibited increased short-term stability, retaining full stability for five days, but also demonstrated increased sfGFP production when compared to fresh CFPS without these additives. This was thought to be a positive consequence of the yield-enhancing properties of MDX and the stability-enhancing properties of β-lactose seen in our previous studies. The role of PEG and its corresponding molecular weight was not clear; however, it does not seem to be as critical as MDX and β-lactose, since no significant difference was detected between the two high-performing formulations (Figure 6c). Their roles could potentially be uncovered through further experimental designs with multiple levels for each factor.

To validate the suitability of the high-performing formulations for sustained room temperature storage, a comprehensive long-term study was undertaken next. Formulation #3 (25 mM MDX, 20 mM β-lactose and 5% PEG 6000) was chosen owing to its highest mean fluorescence and significantly similar outcome to formulation #8 (25 mM MDX, 20 mM β-lactose and 0.5% PEG 600). The experimental setup for the long-term stability study was similar to that for the short-term studies, with the exception of several samples prepared for timed data points up to 2 months. Furthermore, preservation of CFPS was tested on two formats, namely, lyophilised pellets and cellulose stacks (Whatman number 5 filter paper) to evaluate long-term applicability of the two systems. The experimental samples contained formulation #3, and this was tested alongside an unsupplemented sample and a negative control for each format.

**Figure 7:**
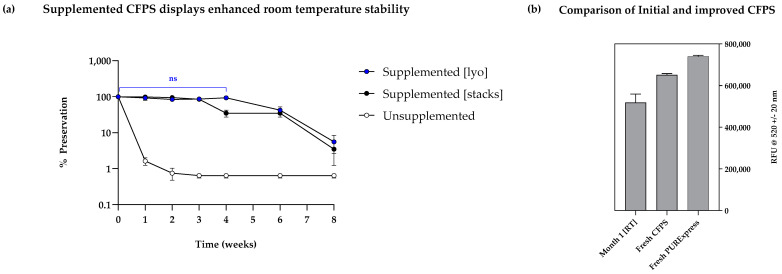
(**a**) Two-month stability study investigating the effects of supplements on sample preservation at room temperature both in lyophilised pellet (‘supplemented [lyo]’) and cellulose stacks (‘supplemented [stacks]’) formats (lyophilised on Whatman number 5 discs) compared with unsupplemented reactions. Supplemented samples were prepared using the standard reaction components, with the addition of the formulation identified from the DoE (25 mM maltodextrin, 20 mM of β-lactose and 0.5% of 6000 MW PEG). Percentage of preservation was calculated by normalising obtained fluorescence values to day 0/week 0 fluorescence values, i.e., samples with no storage; *p*-value > 0.05 = ns. (**b**) Comparison between fresh PURExpress™, fresh in-house CFPS system and rehydrated in-house system supplemented as in (**a**), stored for 1 month at RT. sfGFP fluorescence was normalised to the negative control (pET20b reactions); experiments were performed in triplicates (n = 3); data are shown as means ± SD.

Firstly, a highly fluorescent signal was observed in the lyophilised reactions containing formulation #3, which was sustained from week 0 to week 4. No significant differences were detected in weekly time points from week 0 to week 4, indicating that 100% stability was achieved for one month (one-way ANOVA followed by Tukey’s multiple comparisons test, *p* = 0.8101; Figure 7a). Secondly, samples preserved with formulation #3 on cellulose dropped from 100% to >80% preservation by week 3 and further decreased to ~40% by week 4. Although this result still indicates significantly higher preservation when compared to unsupplemented reactions, which dropped to near 0% preservation by week 1, lyophilised pellets were found to preserve CFPS better than cellulose stacks for one month. However, by week 6, lyophilised pellets and cellulose stacks had significantly similar rates of preservation (<50%), which both dropped to inactive levels by week 8. Collectively, there was a significant increase in the stability of our CFPS system from <1 week to 1+ months, which offers a promising solution for longer utilisation of CFPS on both lyophilised pellets and cellulose stacks. The capabilities of this system could be further increased by storage under vacuum, but the applications for our system are inclusive of transport to extreme environments such as in space and deep-sea vehicles, where this may not be feasible. The DoE approach we have undertaken serves as an efficient initial screening design. If stability beyond one to two months is required, further improvements can be made by executing further DoEs—incorporating a generous sample size and a multi-factorial approach can facilitate better coverage of concentrations, especially for factors such as MDX where only presence or absence was tested.

Whilst the observed output from lyophilised pellets preserved with the new formulation was lower than both fresh in-house and PURExpress^®^ reactions, the stability and yield were both substantially higher than what was previously observed in unprotected CFPS (Figure 7b). This implies that the newly identified formulation functions both as a lyoprotectant and a stabiliser, minimising damage arising from shear forces, light and heat. This systematic approach, guided by DoE, could be used to further advance our understanding of the interplay between additives and CFPS yield and further optimise formulations with improved stability and productivity. Due to the simplicity of the design used, this approach could be easily adapted by other groups to investigate a larger number of factors to further optimise reaction formulation, where stability is of interest. Finally, the cost of our reaction was found to be five times lower than that of PURExpress^®^, making our system more cost-effective and affordable (Supplementary Table S1). The low cost improves accessibility of the technology and the products that are synthesised as a result—the cost of distribution can be further discounted as the system does not require cold storage for at least one month. Collectively, these attributes make the system highly suitable for applications in low-resource environments. With further improvements to stability, applications in extreme environments are hoped to be realised.

## 4. Conclusions

We illustrated an optimised bacterial CFPS system for high-level expression of sfGFP, comparable to highly efficient commercial systems such as PURExpress^®^. Lyophilised pellets and cellulose stack formats were conceptualised for flexible implementation of CFPS in resource-scarce environments, where shelf life and stability of the system were found to be poor. Our strategy of initial screening followed by a minimalistic design of experiments promoted the discovery of formulations with β-lactose and maltodextrin that enhanced sfGFP expression even after five days of room temperature storage when compared to fresh CFPS with no storage. Long-term stability studies revealed 100% preservation of lyophilised CFPS containing this formulation for up to one month, with cellulose stacks displaying > 80%. Thus, we conclude that lyophilised and paper-based systems preserved with the reported formulation contain promising potential for on-demand biomanufacturing and remote biosensing. The success of the formulation underscores the potential of minimalist design of experiments as a powerful tool in optimising and tailoring additives for CFPS systems to enhance productivity and stability.

## Figures and Tables

**Table 1:** Final concentrations of components in CFPS reactions.

Component	Final Concentration	Procurement
*E. coli* cell-free extract (40% *v*/*v*)	40% *v*/*v*	Prepared in-house
Energy components master mix	160 mM HEPES, 4.8 mM ATP sodium salt, 4.8 mM ATP potassium salt, 4.15 mM GTP, 3.2 mM UTP, 3.2 mM CTP, 0.95 mM Coenzyme A, 1.3 mM NAD+, 2.5 mM cAMP, 0.27 mM Folinic acid, 3.3 mM Spermidine, 118.7 mM 3-PGA	Prepared in-house
Amino acid mixture	2.5 mM each	Prepared in-house
Plasmid DNA	10 nM	Prepared in-house
Magnesium glutamate	20 mM	Sigma Aldrich
Potassium glutamate	50 mM	Sigma Aldrich
RNase inhibitor	4 units	Roche
Polyethylene glycol (PEG) 8000	2%	Sigma Aldrich
Nuclease-free water	to 50 μL	Thermo Scientific

**Table 2:** DoE experimental setup consisting of four factors (maltodextrin, MDX; β-lactose; PEG %; and PEG molecular weight, MW) each with two levels (low/high/presence/absence; low/absence highlighted in blue and high/presence highlighted in orange). A formulation without any of the additives was employed as a control run.

Formulation #	MDX (mM)	β-Lactose (mM)	PEG (%)	PEG (MW)
**Control**	0	0	0	n/a
**1**	0	200	0.5	6000
**2**	25	200	5	6000
**3**	25	20	5	6000
**4**	0	20	5	600
**5**	25	200	0.5	6000
**6**	0	20	0.5	6000
**7**	0	200	5	600
**8**	25	20	0.5	600

## Data Availability

The original contributions presented in this study are included in the article/supplementary material. Further inquiries can be directed to the corresponding author.

## Supplementary Materials

The following supporting information can be downloaded at https://www.mdpi.com/article/10.3390/bioengineering12030223/s1: Figure S1: (a): Growth curve of BL21 Star (DE3)—pAR1219 compared with BL21 Star (DE3) shown with and without IPTG induction (n = 6); (b) SDS-PAGE analysis of BL21 Star (DE3) (top) and BL21 Star (DE3)-pAR1219 (bottom). Both soluble and insoluble fractions were collected at the following times: 0 h sample taken before induction; 2 h, 5 h and 24 h samples taken after those hours of induction, respectively. ‘Lad’ indicates molecular ladder. Arrow indicates expected size of T7 RNA polymerase. Induced samples are labelled ‘I’, and soluble samples are highlighted within a grey box. Figure S2: (a) Experimental setup for turbidimetric growth analysis of BL21 Star (DE3)—pAR1219. Each circle indicates one flask. BL21 Star (DE3)—pAR1219 growth curves at (a) 25 °C, (b) 30 °C and (c) 37 °C, respectively. Grey dotted line indicates time of induction, and error bars are smaller than data points, where they are not visible (n = 9; data are shown as means ± SD. Figure S3: SDS-PAGE analysis of (a) soluble and (b) insoluble BL21 Star (DE3)-pAR1219 cell extract obtained from cells grown at 25 °C and induced using varied concentrations of IPTG (0–1 mM IPTG). ‘Lad’ indicates molecular ladder. Figure S4: SDS-PAGE analysis of (a) soluble and (b) insoluble BL21 Star (DE3)-pAR1219 cell extract obtained from cells grown at 30 °C and induced using varied concentrations of IPTG (0–1 mM IPTG). Lad’ indicates molecular ladder. Figure S5: SDS-PAGE analysis of (a) soluble and (b) insoluble BL21 Star (DE3)-pAR1219 cell extract obtained from cells grown at 37 °C and induced using varied concentrations of IPTG (0 mM–1 mM IPTG). Lad indicates molecular ladder. Table S1: Cost breakdown of the reaction components used in this work. Cost per reaction was calculated from price/litre based on value of individually purchased goods. In-house CFPS reactions cost 58p per reaction. Table S2: Cost per litre of CFPS and cost per reaction (50 µL) for in-house CFPS.
