# Supplementary material for: A Design of Experiments Approach for Enhancing Room Temperature Stability of a Lyophilised and Paper-Based Bacterial Cell-Free System
Source: Bioengineering (Basel). 2025 Feb 22;12(3):223. doi: 10.3390/bioengineering12030223 (PMC11939460; doi:10.3390/bioengineering12030223)
Supplement: Supplementary file 1 [file bioengineering-12-00223-s001.zip › bioengineering-3451303-supplementary.pdf]

A Design of Experiments Approach for Enhancing Room Temperature Stability of a Lyophilised and Paper-Based Bacterial Cell-Free System

Tejasvi Shivakumar, Joshua Clark, Alice Goode, Valentine E. Anyanwu and Philip M. Williams *

Molecular Therapeutics and Formulation, School of Pharmacy, University of Nottingham, Nottingham NG7 2RD, UK
*   Correspondence: phil.williams@nottingham.ac.uk

Supplementary Information

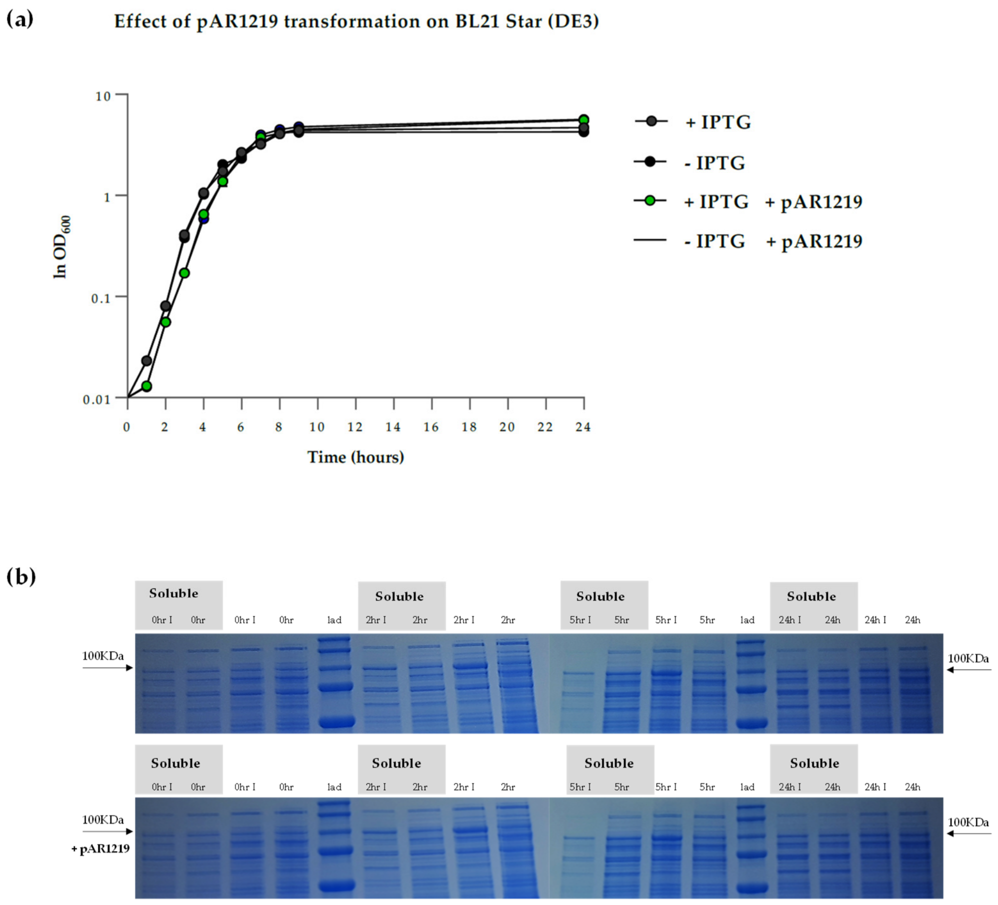

Figure S1 (a): Growth curve of BL21 Star (DE3) – pAR1219 compared with BL21 Star (DE3) shown with and without IPTG induction (n = 6); (b) SDS-Page analysis of BL21 Star (DE3) (top) and BL21 Star (DE3)-pAR1219 (bottom). Both soluble and insoluble fractions were collected in the following times: 0 hr sample taken before induction; 2 hr, 5 hr and 24 hr samples taken after those hours of induction respectively. 'Lad' indicates molecular ladder. Arrow indicates expected size of T7 RNA polymerase. Induced samples are labelled 'I' and soluble sample are highlighted within a grey box.

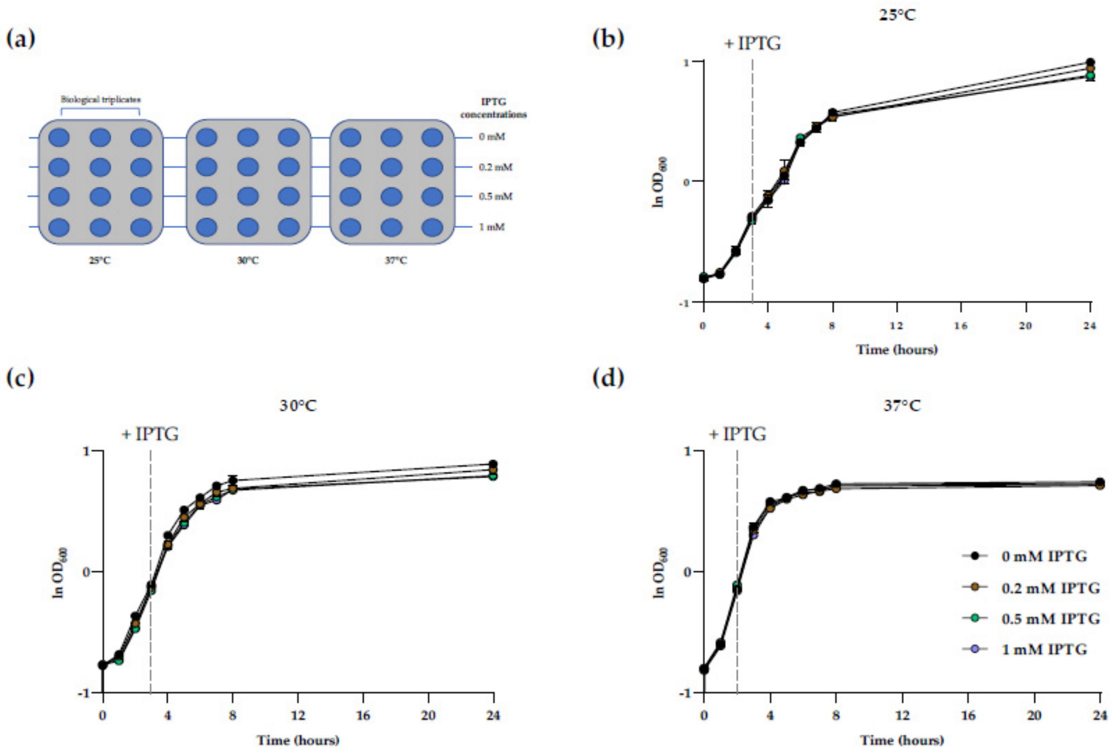

Figure S2: (a) Experimental set up for turbidimetric growth analysis of BL21 Star (DE3) – pAR1219. Each circle indicates 1 flask. BL21 Star (DE3) - pAR1219 growth curves at (a) 25°C, (b) 30°C and (c) 37°C respectively. Grey dotted line indicates time of induction and error bars are smaller than data points, where they are not visible (n = 9; Data are shown as mean ± SD.

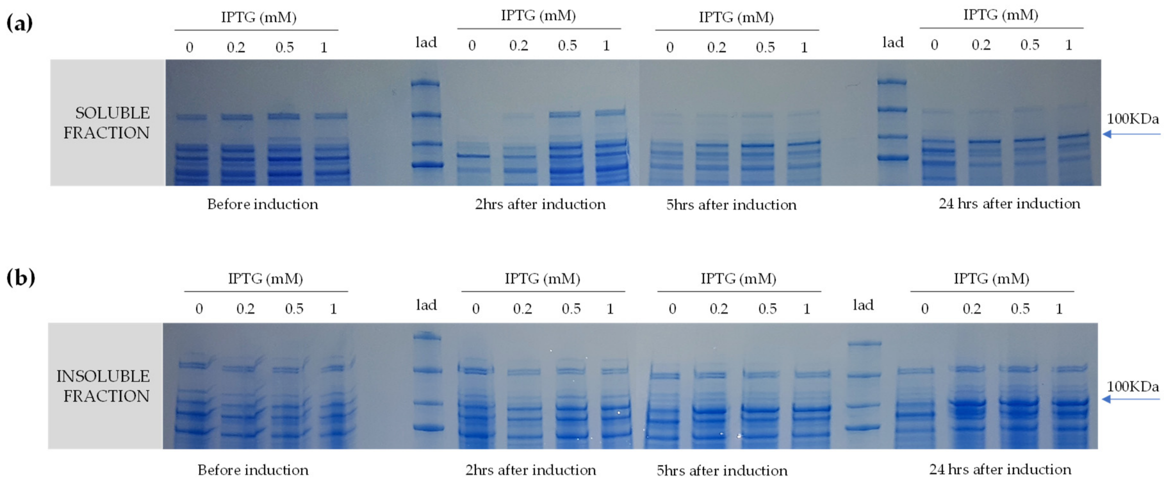

Figure S3: SDS-Page analysis of (a) soluble and (b) insoluble BL21 Star (DE3)-pAR1219 cell extract obtained from cells grown at 25°C and induced using varied concentrations of IPTG (0 – 1 mM IPTG). 'Lad' indicates molecular ladder.

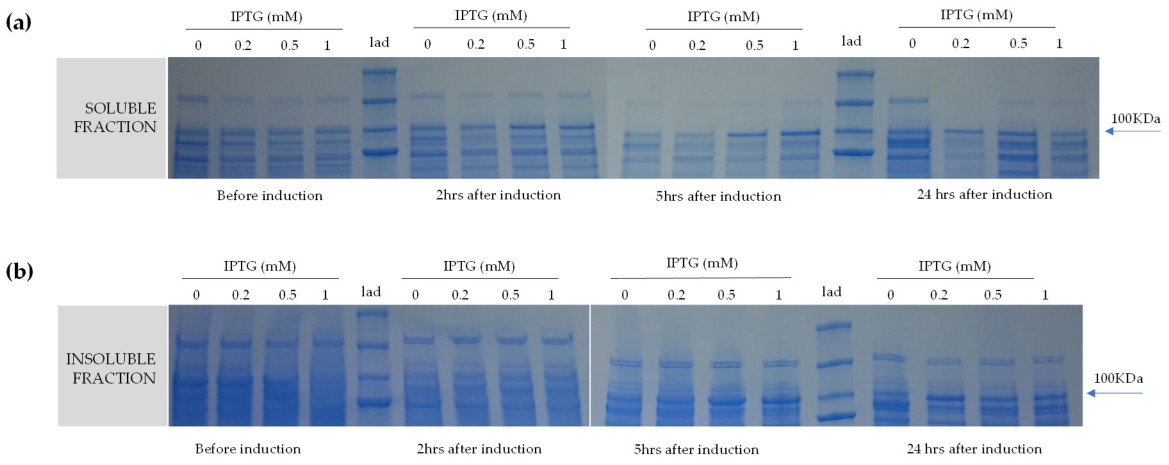

Figure S4: SDS-Page analysis of (a) soluble and (b) insoluble BL21 Star (DE3)-pAR1219 cell extract obtained from cells grown at 30°C and induced using varied concentrations of IPTG (0 – 1 mM IPTG). Lad' indicates molecular ladder.

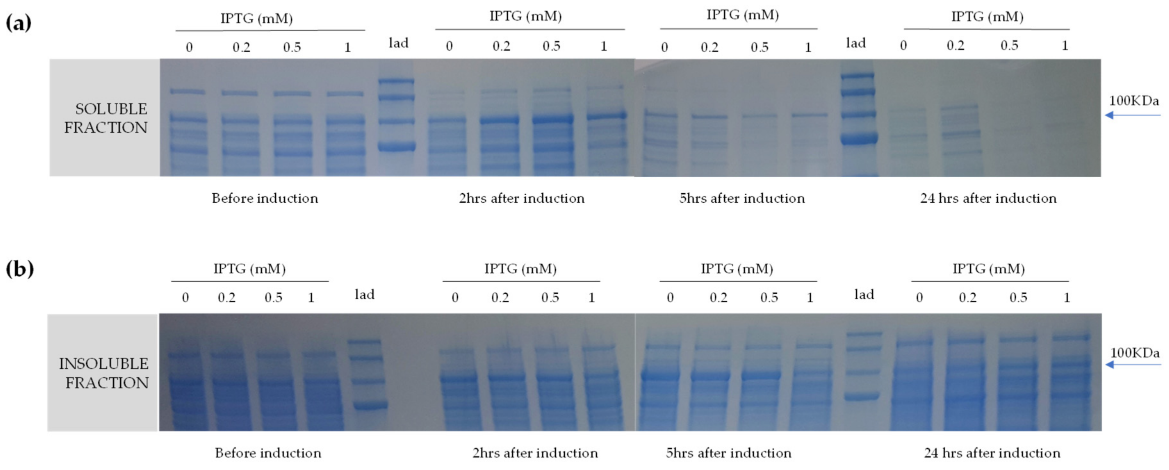

Figure S5: SDS-Page analysis of (a) soluble and (b) insoluble BL21 Star (DE3)-pAR1219 cell extract obtained from cells grown at 37°C and induced using varied concentrations of IPTG (0 mM – 1 mM IPTG). Lad' indicates molecular ladder.

Synthetic DNA fragments (sfGFP-His):

TAATACGACTCACTATAGGGAGACCACAACGGTTTCCCTCTAGAAATAATTTTGTTTAACTTT
AAGAAGGAGATATACATATGCGTAAAGGCGAAGAGCTGTTCACTGGTGTCGTCCCTATTCTG
GTGGAACTGGATGGTGATGTCAACGGTCATAAGTTTTCCGTGCGTGGCGAGGGTGAAGGTGA
CGCAACTAATGGTAAACTGACGCTGAAGTTCATCTGTACTACTGGTAAACTGCCGGTACCTTG
GCCGACTCTGGTAACGACGCTGACTTATGGTGTTCAGTGCTTTGCTCGTTATCCGGACCATATG
AAGCAGCATGACTTCTTCAAGTCCGCCATGCCGGAAGGCTATGTGCAGGAACGCACGATTTC
CTTTAAGGATGACGGCACGTACAAAACGCGTGCGGAAGTGAAATTTGAAGGCGATACCCTG
GTAAACCGCATTGAGCTGAAAGGCATTGACTTTAAAGAAGACGGCAATATCCTGGGCCATAA
GCTGGAATACAATTTTAACAGCCACAATGTTTACATCACCGCCGATAAACAAAAAAATGGCA
TTAAAGCGAATTTTAAAATTCGCCACAACGTGGAGGATGGCAGCGTGCAGCTGGCTGATCAC
TACCAGCAAAACACTCCAATCGGTGATGGTCCTGTTCTGCTGCCAGACAATCACTATCTGAGC
ACGCAAAGCGTTCTGTCTAAAGATCCGAACGAGAAACGCGATCATATGGTTCTGCTGGAGTT
CGTAACCGCAGCGGGCATCACGCATGGTATGGATGAACTGTACAAACACCACCACCACCAC
CACTGA

Forward primer: actttaagaaggagatatacatatgcgtaaaggcgaagagctgttc
Reverse primer: tttgtacagttcatccataccatgcgtgatg

Cost analysis

Table S1: Cost breakdown of the reaction components used in this work. Cost per reaction was calculated from price/litre based on value of individually purchased goods. In-house CFPS reactions costed 58p per reaction.

Component Name	Vendor	Category #	£/g	g/L	£/L	uL/58.7uL	£/L of Reacti
K-Glu (1M)	Sigma-Aldrich/Merck	G1149	£0.33	203.23	£67.33	2.50	£2.87
Mg-Glu (1M)	Sigma-Aldrich/Merck	49605	£0.17	388.61	£64.68	1.00	£1.10
Maltodextrin (0.285M)	Sigma-Aldrich/Merck	419672	£0.28	302.50	£83.80	2.10	£3.00
B-Lactose (2.5M)	Sigma-Aldrich/Merck	L3750	£0.45	855.75	£380.89	0.40	£2.60
PEG 6000 (50%v/v)	Sigma-Aldrich/Merck	P2139	£0.04	500.00	£22.16	5.00	£1.89
RNAse In	Roche/Sigma-Aldrich	3335399001	N/A	N/A	£1,120,000.00	0.20	£3,816.01
Energy					£44,144.42	8.00	£6,016.28
HEPES	Sigma-Aldrich/Merck	H4034	£0.03	N/A	7.56	N/A	N/A
Adenosine 5′-triphosphate disodiu	Sigma-Aldrich/Merck	A7699	£27.29	N/A	453.05	N/A	N/A
Adenosine 5′-triphosphate dipotas	Sigma-Aldrich/Merck	A8937	£41.45	N/A	729.45	N/A	N/A
GTP	Sigma-Aldrich/Merck	G8877	£119.52	N/A	1625.47	N/A	N/A
UTP	Sigma-Aldrich/Merck	U6750	£116.77	N/A	1284.47	N/A	N/A
CTP	ThermoFisher Scientific	J14121-03	£294.10	N/A	3117.46	N/A	N/A
CoA	Sigma-Aldrich/Merck	C4780	£2,955.30	N/A	13594.38	N/A	N/A
NAD	Sigma-Aldrich/Merck	N6522	£62.64	N/A	338.25	N/A	N/A
cAMP	Sigma-Aldrich/Merck	A9501	£151.74	N/A	789.06	N/A	N/A
Folinic Acid	Sigma-Aldrich/Merck	47612	£292.94	N/A	234.35	N/A	N/A
Spermidine	Sigma-Aldrich/Merck	85558	£28.04	N/A	84.12	N/A	N/A
3-Phosphoglyceric acid	Sigma-Aldrich/Merck	P8877	£158.60	N/A	21886.80	N/A	N/A
Amino acids					£65.26	12.50	£13.90
L-Alanine	Sigma-Aldrich/Merck	05129	£1.07	N/A	0.95	N/A	N/A
L-Arginine	Sigma-Aldrich/Merck	11009-25g-F	£1.08	N/A	1.88	N/A	N/A
L-Aspartic acid	Sigma-Aldrich/Merck	11189	£0.25	N/A	0.33	N/A	N/A
L-Asparagine	Sigma-Aldrich/Merck	A4159	£0.39	N/A	0.52	N/A	N/A
L-Glutamine	Sigma-Aldrich/Merck	49419	£0.83	N/A	1.21	N/A	N/A
L-Glutamic acid	Sigma-Aldrich/Merck	49449	£0.18	N/A	0.26	N/A	N/A
L-Glycine	Sigma-Aldrich/Merck	50046	£0.12	N/A	0.09	N/A	N/A
L-Histidine	Sigma-Aldrich/Merck	53319	£1.51	N/A	2.35	N/A	N/A
L-Isoleucine	Sigma-Aldrich/Merck	58879	£2.44	N/A	3.19	N/A	N/A
L-Lysine	Sigma-Aldrich/Merck	L5501	£1.77	N/A	2.59	N/A	N/A
L-Methionine	Sigma-Aldrich/Merck	64319-25g-F	£1.72	N/A	2.56	N/A	N/A
L-Phenylalanine	Sigma-Aldrich/Merck	78019	£1.10	N/A	1.81	N/A	N/A
L-Proline	Sigma-Aldrich/Merck	81709	£2.00	N/A	2.32	N/A	N/A
L-Serine	Sigma-Aldrich/Merck	84959	£2.51	N/A	2.65	N/A	N/A
L-Threonine	Sigma-Aldrich/Merck	89179	£1.85	N/A	2.21	N/A	N/A
L-Valine	Sigma-Aldrich/Merck	94619	£1.85	N/A	2.15	N/A	N/A
L-Tryptophan	Sigma-Aldrich/Merck	93659	£2.80	N/A	5.72	N/A	N/A
L-Tyrosine	Sigma-Aldrich/Merck	93829	£1.28	N/A	2.31	N/A	N/A
L-Leucine	Sigma-Aldrich/Merck	61819	£0.97	N/A	1.27	N/A	N/A
L-Cysteine	Sigma-Aldrich/Merck	30089	£1.20	N/A	1.45	N/A	N/A
KOH (2M)	Sigma-Aldrich/Merck	P5958	£0.07	N/A	27.43	N/A	N/A
Extract					£4.67	22.00	£1.75
IPTG (1M)	VWR	437145X	£2.93	0.24	£0.70	N/A	N/A
Ampicillin (100mg/mL)	Sigma-Aldrich/Merck	A951	£2.24	0.10	£0.22	N/A	N/A
D-Glucose (0.4M)	Sigma-Aldrich/Merck	G8270	£0.01	18.00	£0.12	N/A	N/A
NaCl	Sigma-Aldrich/Merck	S3014	£0.01	5.00	£0.07	N/A	N/A
Tryptone	Scientific Laboratory Supplie	LP0042B	£0.12	16.00	£1.89	N/A	N/A
Yeast Extract	Fisher Scientific UK	LP0021B	£0.09	10.00	£0.92	N/A	N/A
Potassium Phosphate Monobasic	Fisher Scientific UK	424205000	£0.06	7.00	£0.39	N/A	N/A
Potassium Phosphate Dibasic	Sigma-Aldrich/Merck	P8281	£0.12	3.00	£0.36	N/A	N/A
DNA			**Per 1 preparation**		£16.52	5.00	£1.41
QIAGEN Plasmid Plus Maxi Kit (100	Qiagen	12965	£16.52				

**Total £/L of In-House Reaction:**	£9,860.79
**Total £/Reaction of In-House:**	£0.58

Table S2: Cost per litre of CFPS and cost per reaction (50 μl) for in-house CFPS
